# Developing a guidance resource for managing delirium in patients with COVID-19

**DOI:** 10.1017/ipm.2020.71

**Published:** 2020-05-28

**Authors:** David Meagher, Dimitrios Adamis, Suzanne Timmons, Niamh A O’Regan, Shaun O’Keeffe, Sean Kennelly, Catherine Corby, Anna Maria Meaney, Paul Reynolds, Mas Mohamad, Kevin Glynn, Roisin O’Sullivan

**Affiliations:** 1 Department of Psychiatry, University of Limerick School of Medicine, Limerick, Ireland; 2 Sligo-Leitrim Mental Health Services, Ballytivan Rd, Sligo, Ireland; 3 Geriatric Medicine, University College Cork, Centre for Gerontology and Rehabilitation, St. Finbarr’s Hospital, Cork, Ireland; 4 Geriatric Medicine, Waterford University Hospital, Waterford, Ireland; 5 Geriatric and Stroke Medicine, Tallaght University Hospital Memory Assessment Service, Tallaght University Hospital, Dublin; 6 CL Psychiatry, University Hospital Limerick, Limerick, Ireland; 7 Psychiatry for Later Life, University Hospital Limerick, Limerick, Ireland; 8 Perinatal Psychiatry, University Hospital Limerick, Limerick, Ireland

**Keywords:** COVID-19, delirium, guidance

## Abstract

As the COVID-19 pandemic escalates worldwide, it is apparent that many patients with more severe illness will also experience delirium. These patients pose a particular challenge in the application of optimal care due to issues with infectious risk, respiratory compromise and potential interactions between medications that can be used to manage delirium with antiviral and other treatments used for COVID-19. We describe a guidance resource adapted from existing guidelines for delirium management that has been tailored to the specific challenge of managing delirium in patients with COVID-19 infection. Issues around the assessment and treatment of these patients are examined and distilled into a simple (one-paged guidance resource that can assist clinicians in managing suspected delirium.

## Background

The healthcare community is currently in the grip of a pandemic due to COVID-19 (coronavirus SARS-CoV-2) (Zhou *et al.*, 2020), with growing worldwide mortality especially amongst elderly and those with pre-existing comorbidities, such as cardiorespiratory disease, diabetes and dementia. In addition to fever and respiratory symptoms, a substantial number of patients experience neurological difficulties, with reports of ‘impaired consciousness’ in 15% of those with severe illness in one series (Mao *et al.*, 2020) and ‘confusion’ reported in 9% at presentation in another (Chen *et al.*, 2020; Meo *et al.*, 2020).

Against this backdrop, it can be expected that delirium will complicate illness course in many patients with COVID-19. Moreover, the management of delirium in these patients is especially challenging as the application of many non-pharmacological strategies to manage delirium is curtailed by the need to minimise infectious risk (LaHue *et al.*, 2020), while medications used in delirium management have recognised capacity to cause respiratory depression and have interactions with antiviral and other agents used to treat COVID-19.

As a result, existing guidelines on delirium management need to be carefully considered and adapted to the needs of patients with COVID-19. From an Irish perspective, clinicians from the Department of Psychiatry at University Hospital Limerick have with psychiatry and geriatric medicine clinicians that have particular interest in the management of delirium to develop a user-friendly and practical guidance document that is tailored to the particular challenge of suspected delirium in patients with COVID-19. In this paper, we describe the development of this resource and examine emerging literature that addresses key considerations relevant to the provision of optimal care to patients with COVID-19 who experience delirium.

## Existing resources

The management of delirium in everyday clinical practice is typically guided by a variety of existing resources that include formal guidelines (e.g. National Institute for Health and Clinical Excellence, 2010; Scottish Intercollegiate Guidelines Network, 2019), as well as various guidance material developed by local departments (e.g. Policy for management of suspected delirium Psychiatry for later Life service, University Hospital Limerick) as well as by National groups (e.g. Health Services Executive: early identification and management of delirium in the emergency department and acute medical assessment unit). These resources address many important aspects of delirium as it relates to the legion of possible causes that occur in everyday clinical practice including detection and diagnosis, investigation for underlying causes, non-pharmacological management and advice regarding the circumstances under which pharmacological interventions can be applied, including choice of agent, dosing and monitoring of response and adverse effects.

## Additional guidance resources

In response to the COVID-19 pandemic, a variety of position statements and guidance resources documents have emerged to supplement these existing resources by addressing the specific challenges posed by patients with COVID-19 in terms of minimising infectious risk through efficient recognition of delirium, prudent application of environmental and other non-pharmacological efforts to minimise the occurrence and impact of delirium in COVID-19 patients, and the key considerations around use of pharmacological interventions, including their rationale, interactions with other medications that may be used in these patients and potential for adverse effects (British Geriatrics Society, European Delirium Association, Old Age Psychiatry Faculty of the Royal College of Psychiatrists, 2020; Gee and Taylor, 2020; Liverpool Drug Interactions Group).

## Detection

A number of issues particular to delirium assessment in patients with COVID-19 are evident. Firstly, delirium detection must be sensitive to the need for rapid and efficient assessment that minimises the duration of interactions that can be physically and mentally demanding on highly morbid patients. The 4AT is a practical and simple tool for the efficient assessment of possible delirium that is brief (requires less than 2 minutes), has excellent patient coverage (i.e. allows assessment of patients with severe drowsiness or agitation who are less able to communicate) and does not require any special training. It is supported by at least 11 validation studies (involving >2500 patients) that indicate high sensitivity (83–100%) with moderate to high specificity (70–99%) for delirium (seewww.4AT.com) (Shenkin *et al.*, 2019). For these reasons, the 4AT has become the preferred tool that is recommended for delirium detection by recent guidelines and is suited to the challenges of COVID-19 as it is brief, minimally demanding upon patients and does not include any elements (e.g. pen and paper) that require physical contact. It is important to note that where a patient is unable to engage with testing (e.g. the months backwards test), this is considered a failed performance and scored accordingly. This reduces the likelihood of patients with severe delirium being missed.

## Non-pharmacological management

Early reports from the Italian experience of the COVID-19 pandemic (di Giacomo *et al.*, 2020) have highlighted how providing a delirium-friendly care environment is extremely challenging for many patients with COVID-19 as for many the experience of being nursed in isolation by staff using Personal Protective Equipment (PPE) may create an anxiogenic and threatening care environment. In addition, many of the principles of routine management of the care environment may not be realistic, such as consistency of staffing, facilitated mobilisation, providing bedside sitters and involving family in care provision. However, many of the other elements to good care of those at risk of delirium continue to apply, such as optimising sensory abilities, clear and concise communication and careful attention to medication regimes to minimise use of deliriogenic medications and polypharmacy. In addition, in many centres, staff have identified creative ways of reducing the impersonal nature of providing care when using PPE, such as wearing large named identification photographs when engaging with patients receiving care in isolation.

Outbreaks of COVID-19 have become common in residential care settings in Ireland, as elsewhere, and in many cases delirium can be the principal presenting feature. Many such residents are frail, and some are approaching the end of life. It is usually appropriate to manage such residents in the nursing home, often with palliative care measures, unless it is judged that transfer an acute hospital may provide clinical benefit. This presents a challenging risk-benefit analysis that requires careful consideration of baseline functioning and the likely benefits of more intense supportive intervention versus the recognised risk of provoking or exacerbating delirium already delirium-prone persons. The detection and management strategies outlined in this paper can also be applied in nursing home settings, with adaptation according to the resources that are available in each setting.

## Assessing causation

Delirium can be secondary to insults located within the Central Nervous System (CNS) but also commonly occurs in response to disturbances that are primarily located systemically, such as peripheral infection, organ failure or metabolic disruptions. The precise mechanisms by which COVID-19 may cause neurological manifestations are still unclear but may include direct CNS infection, access due to reduced blood–brain barrier integrity, retrograde neuronal transport, hypoxic damage, vascular mechanisms and neuroinflammatory responses, along with the many other causes that have been associated with increased delirium propensity. In addition, patients in isolation, requiring mechanical ventilation, with reduced sensory input and mobilisation are all more prone to developing delirium (Kotfis *et al.*, 2020).

**Figure 1.**
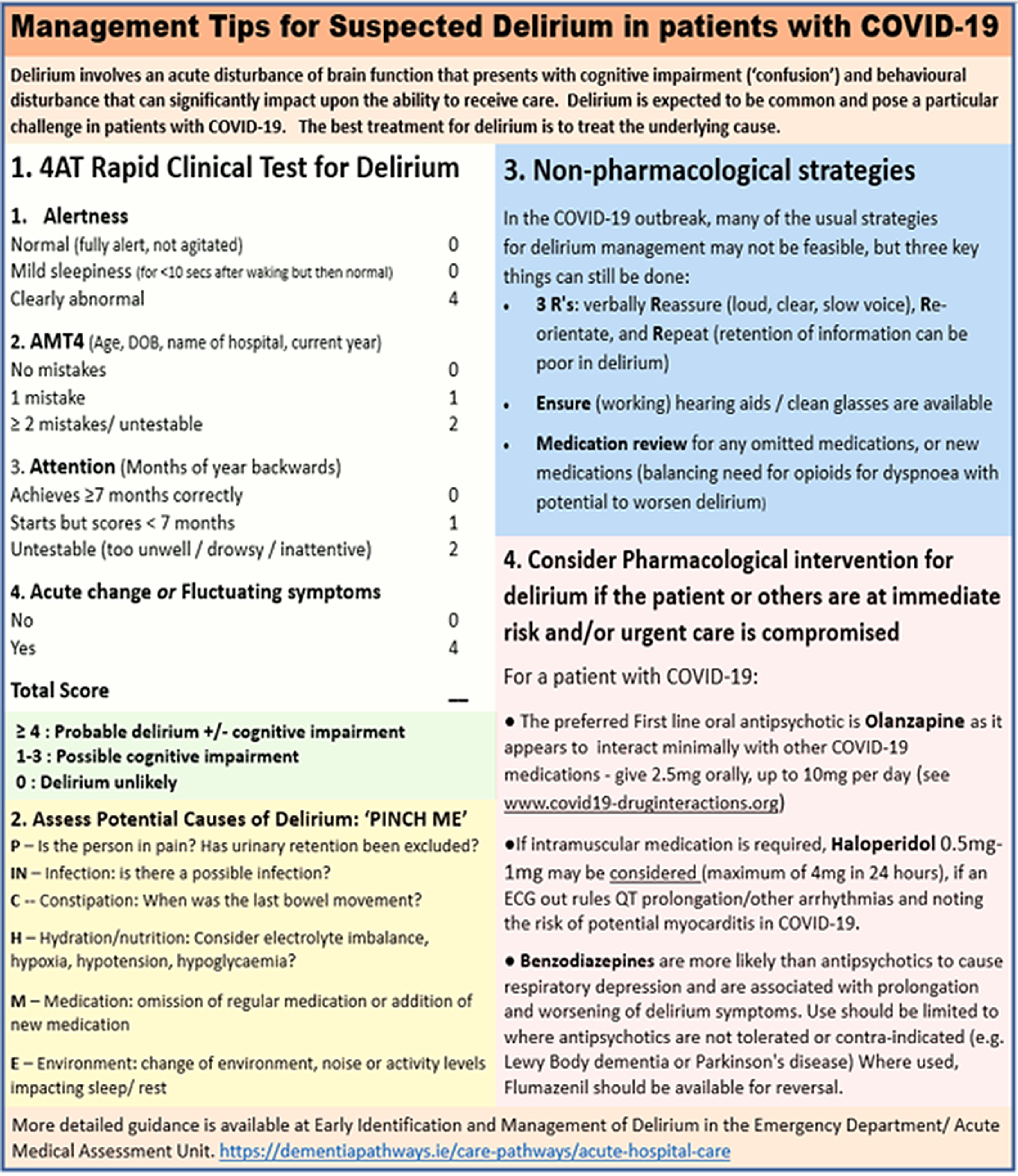
Management tips for suspected delirium in patients with COVID-19.

In addressing delirium, it is always important to recognise that it is a multifactorial condition, typically with more than one causative factor and that a variety of factors can serve as precipitating and/or aggravating issues. The primary aim of delirium treatment is to address the aetiological cause(s). The PINCH-ME algorithm (see Fig. 1) is frequently used to guide aetiological assessment and is applicable to patients with COVID-19.

## Management of agitated and distressed patients with delirium

In addition, the delirious state can be highly problematic in terms of patient distress and behavioural disturbance. The use of antipsychotic agents in the management of delirium is the source of ongoing debate with the current consensus that these agents are not a treatment for delirium *per se* (Burry *et al.*, 2018; Nikooie *et al.*, 2019) but can be used to manage symptoms of delirium where the balance of risk in terms of potential adverse effects allows (Meagher *et al.*, 2018). Although the demographic pattern of older patients being prone to experiencing more severe illness with COVID-19 suggests that hypoactive presentations will be prominent (Meagher, 2009), a substantial minority will experience hyperactivity that impacts upon patient wellbeing in terms of distressing psychotic and affective disturbances, risk of falls and other injuries, ability to receive optimal care and elevated infectious risk. It may be expected that with reduced capacity to provide non-pharmacological supports, medications will need to be considered in a substantial percentage of these patients. However, these decisions are further complicated by heightened vulnerability to adverse effects from pharmacological treatments. In addition to the usual concerns about toxicity in terms of extrapyramidal effects, cardiotoxicity and cerebrovascular effects, patients with COVID-19 can be expected to be more vulnerable to respiratory depression and cardiac effects.

## Benzodiazepines

Although there is no evidence to support the use of benzodiazepines in the treatment of delirium among hospitalised patients except in alcohol or benzodiazepine withdrawal (Lonergan *et al.*, 2009) or in palliative care (Finucane et al 2020), they are still in use despite their documented capacity to cause or worsen delirium. Both antipsychotic agents and benzodiazepines have potential to cause respiratory depression, but this is particularly significant with benzodiazepines which are associated with dose-related centrally mediated respiratory depression (Ekstrom *et al.*, 2014; Vozoris, 2014) and, as such, benzodiazepines should only be used with great caution in patients with respiratory compromise (Shah *et al.*, 2017). In summary, benzodiazepine use should be avoided for treating delirium in COVID-19 infected patients (LaHue *et al.*, 2020) or should be limited to patients who cannot tolerate antipsychotics or who have other contraindications to their use (e.g. Parkinson’s disease or Lewy body dementia) or have withdrawal or seizure-related symptoms. Where benzodiazepines are used, this should include careful monitoring of effects and an awareness that their respiratory effects can be reversed with flumazenil. It must equally be highlighted that benzodiazepines are perfectly appropriate as a palliative treatment for severe respiratory distress, including end-of-life care, and indeed have an important role here in palliation and reducing anxiety. This indication is clearly differentiated from their use specifically to treat delirium and/or worsened responsive behaviours.

## Antipsychotic agents

As such, where pharmacological treatment is required to counter the challenge of distressing psychosis and/or otherwise unmanageable behavioural disturbance, antipsychotic agents are considered the first choice intervention. Existing guidelines varying in suggested agents of first choice, with haloperidol, olanzapine, risperidone and quetiapine recommended as possible treatments. However, in the context of COVID-19, cardiac effects, particularly when used in combination with antiviral agents, are an important concern and evidence suggests that olanzapine has a favourable profile compared to risperidone and quetiapine (see below), while haloperidol remains a useful option due to the range of routes by which it can be administered (see Table 1).

**Table 1.** Drug interactions between commonly used medications in delirium and COVID-19 agents (adapted from Liverpool drug interactions group)

	ATZ	LPRT	REM	FAV	CHL	NIT	RIB	TOC
Haloperidol	↑♥	↑♥	↔	↔	↔♥	↔	↔	↔
Olanzapine	↔	↓	↔	↔	↔	↔	↔	↔
Risperidone	↑♥	↑♥	↔	↔	↑♥	↔	↔	↔
Quetiapine	↑♥	↑♥	↔	↔	↔♥	↔	↔	↔
Diazepam	↑	↑	↔	↔	↔	↔	↔	↔
Lorazepam	↔	↔	↔	↔	↔	↔	↔	↔
Midazolam	↑	↑	↔	↔	↔	↔	↔	↔

ATZ, atazanavir; LPRT, lopinavir/ritonavir; REM, remdesivir; FAV, favipiravir; CHL, chloroquine/hydroxychloroquine; NIT, nitazonide; RIB, ribavirin; TOC, tocilizumab.

↑ indicates potential for increased medication effects and ↓ indicates potential for decreased medication effects. ♥ indicates potential cardiac toxicity that may cause QT and/or PR prolongation.

Where antipsychotic agents are used, it is important to monitor cardiac function and in particular to rule out QT prolongation with a baseline Electrocardiogram (ECG). Moreover, use of antipsychotics brings with it a risk of a variety of other potential adverse effects, with a significantly increased risk of cerebrovascular incidents especially in patients with pre-existing cognitive issues, such as dementia (Rao *et al.*, 2016), as well as extrapyramidal and anticholinergic effects (e.g. cardiac conduction effects and increased hyperpyrexia risk), with the latter more commonly attributed to olanzapine than other second generation antipsychotics (Gardner *et al.*, 2005). Of note, the evidence suggests that extrapyramidal effects are uncommon with low dose use (Burry *et al.*, 2018). Other evidence suggests that cardiac effects are rare where cumulative daily doses of intravenous haloperidol are lower than 2 mg, unless patients have additional risk factors for QTc prolongation (Meyer-Massetti *et al.*, 2010). Suggested doses are shown in Figure 1 with the usual rule of ‘start low and go slow’ particularly important given the age profile and level of morbidity of many patients with symptomatic COVID-19. In addition, patients experiencing hyperinflammatory states can have increased brain permeability to neurotoxins that in turn can confer greater sensitivity to adverse effects from psychotropic agents (Wu *et al.*, 2020).

## Potential interactions between psychotropic and antiviral agents

A further consideration relates to potential interactions between psychotropic agents and other treatments used in these patients, with antiviral agents a particular focus of concern. The Liverpool Drug Interaction Group (based at the University of Liverpool, UK), in collaboration with the University Hospital of Basel (Switzerland) and Radboud UMC (Netherlands), has collated information regarding interactions between over 400 medications (including psychotropics) and experimental COVID-19 therapies (e.g. atazanavir, lopinavir/ritonavir, remdesivir, favipiravir, chloroquine, hydroxychloroquine, ribavirin, tocilizumab, interferon beta)(see www.covid19-druginteractions.org). This information indicates a favourable profile for olanzapine in terms of interactions with antiviral agents, while haloperidol, risperidone and quetiapine increase the exposure to potential adverse effects of many antiviral agents and haloperidol warrants particular caution in respect of potential for effects on cardiac conduction.

## A guidance resource for managing delirium in COVID-19 patients

Taking all of these considerations into account, we have composed a guidance sheet that can assist non-expert clinicians in how to manage COVID-19 patients that have suspected delirium. It is brief (occupying one side of a page) and practical, addressing four steps in decision-making: (1) assessment for delirium using the 4AT, (2) assessing potential aetiological factors using the PINCH-ME algorithm, (3) guidance on non-pharmacological management and finally, (4) guidance on use of pharmacological interventions. While in many cases, clinicians will be comfortable in detecting and managing delirium, this guidance can assist where a more structured approach is needed. It can also serve as a useful support to guide efforts to assess and manage delirium in consultation with psychiatry services. This guidance provides a rapid response to the need to focus our efforts to manage delirium during the pandemic that has been disseminated to support everyday practice in local services and beyond. It can also provide a document that can be further developed in a more systematic way (e.g. consensus guidelines) over time as further evidence (e.g. around existing or additional treatments for COVID-19) emerges.
